# 10-year risk for cardiovascular diseases using WHO prediction chart: findings from the civil servants in South-western Nigeria

**DOI:** 10.1186/s12872-020-01438-9

**Published:** 2020-03-31

**Authors:** Olaniyan Akintunde Babatunde, Sunday Olakunle Olarewaju, Adeleye Abiodun Adeomi, Joel Olufunminiyi Akande, Adebobola Bashorun, Chukwuma David Umeokonkwo, James Olusegun Bamidele

**Affiliations:** 1 Department of Community Medicine, Ladoke Akintola University of Technology, Ogbomoso, Oyo State Nigeria; 2 Nigeria Field Epidemiology and Laboratory Training Program, Abuja, Nigeria; 3 Oyo State Primary Healthcare Board, Agodi, Ibadan, Oyo State Nigeria; 4 Department of Community Medicine, Osun State University, Osogbo, Osun State Nigeria; 5 Department of Community Medicine, Obafemi Awolowo University, Ile-Ife, Osun State Nigeria; 6 Department of Chemical Pathology, BOWEN University Teaching Hospital, Ogbomoso, Oyo State Nigeria; 7 African Field Epidemiology Network, Abuja, Nigeria; 8 Department of Community Medicine, Alex Ekwueme Federal University Teaching Hospital, Abakaliki, Ebonyi State Nigeria; 9 Department of Community Medicine, Ekiti State University, Ado-Ekiti, Ekiti State Nigeria

**Keywords:** Cardiovascular diseases, Risk factors, Prevalence, Prediction chart, Civil servants

## Abstract

**Background:**

Globally, cardiovascular diseases (CVDs) have continued to ravage the human existence through the premature deaths of its workforce. Despite this burden, many studies in Nigeria have focused on determining the prevalence of risk factors which alone are insufficient to assess the risk of future cardiovascular events. Therefore, we determined the pattern and predictors of 10-year risk for CVDs in South-western Nigeria.

**Methods:**

We conducted a cross-sectional study among workers at the local government areas (LGAs) of Oyo State. Using a multi-stage sampling technique, we recruited 260 respondents from the LGA secretariats. A pre-tested, interviewer-administered questionnaire was administered to obtain information on the socio-demographics and behavioural attributes. Lipid analysis, anthropometric, blood pressure, fasting blood glucose measurements were done using standard protocols. The respondents’ CVD risk was assessed using WHO prediction chart. Data were analyzed using IBM SPSS version 25; bivariate analysis was done using Chi-square and binary logistic regression was used to identify the predictors of 10-year risk for CVDs at 5% level of significance.

**Results:**

The mean age of respondents was 46.0 + 6.7 years. The proportion of respondents with good knowledge of risk factors was 57.7%. The prevalence of CVD risk factors were as follows: systolic hypertension (29.6%), visceral obesity (35.8%), diabetes mellitus (18.8%), smoking (5.8%), elevated total cholesterol (55.4%) and physical inactivity (84.6%). The proportion of respondents with low, moderate and high risk of developing CVDs within 10 years was 76.9, 8.5 and 14.6% respectively. Respondents with age ≥ 40 years (aOR = 2.6, 95% CI = 1.3–8.5), management cadre (aOR = 3.8, 95% CI = 1.6–9.6), obesity (aOR = 4.8, 95% CI = 1.2–120), abnormal waist circumference (aOR = 2.8, 95% CI = 1.3–5.2) and physical inactivity (aOR = 2.4, 95% CI = 1.2–4.7) were associated with the higher likelihood of developing CVDs.

**Conclusion:**

About one-sixth of the respondents had high risk of developing CVDs within the next 10 years and it is likely that it will reduce the productivity of the State. Lifestyle modification and early detection of risk factors through regular screening programmes for those with high CVD risk is therefore recommended.

## Background

Cardiovascular diseases (CVDs) are known as a group of heart and blood vessel-related disorders [1]. Globally, CVDs have continued to ravage the existence of human race and equally threatened the productive sector of many countries through premature deaths of their workforce [2]. It remains a disease of public health importance due to the attendant high morbidity and mortality [3]. Worldwide, CVDs are the number one cause of death as more people die annually from it than any other causes [3, 4]. In 2015, about 17 million premature deaths occur in global south due to non-communicable disease (NCD), of which 37% were caused by CVDs. In 2016, an estimated 17.9 million people died from CVDs, representing 31% of all global deaths [3, 4]. More than three-quarters of the global deaths from CVDs occur in developing nations [5].

Hitherto, the health system in the developed world was greatly challenged with these high burdens of CVDs and its complications, but in recent times, there was a reversal of this ugly trend due to medical advances, improved healthcare delivery and good health-seeking behaviours [6]. However, in the global south, there is a significant rise in the incidence of CVDs resulting from epidemiological transition, increased urbanization and poor knowledge of risk factors for CVDs [7, 8]. This has resulted in an increased mortalities among the working populations [9].

The recent rise in CVDs in developing countries, including Nigeria has almost reached epidemic proportion [10] and this has led to a renewed effort to reinforce the use of affordable preventive strategies to stem this tide. For example, CVDs accounted for estimated 12% of all deaths in Nigeria [11] and a large percentage of medical admissions in a teaching hospital in south western part of Nigeria were due to CVDs [12]. Consequent upon this, WHO has identified very cost-effective interventions that are implementable even in low-resource settings for prevention and control of CVDs [5]. More importantly, these interventions need to be targeted at those with high total cardiovascular risk or those with single risk factor levels above traditional thresholds such as hypertension and hypercholesterolemia.

To determine the level of CVD risk in the individuals, the strategy involves the use of CVD risk calculators, such as the World Health Organization/International Society of Hypertension (WHO/ISH) prediction charts [5], Framingham [13], etc. Many studies have found the usefulness of these charts in quantifying individual’s risk of developing CVDs in the next 10 years [14, 15]. Their use is important in making informed decisions about the type and course of management to be instituted, especially in the low-resource settings, where skilled manpower, such as cardiologists is in short supply [16]. This prediction chart is found to be cost-effective and assess the total cardiovascular risk through the integration of risk factors (age, sex, presence or absence of diabetes, smoking status, systolic blood pressure, total serum cholesterol). Interestingly, this tool could be handled by clinicians and other health workers to prevent cardiovascular events (especially, stroke and heart attacks) in the developing countries [17].

Developing countries are faced with a double burden of diseases, lack of awareness of CVD predictors and huge health-infrastructural deficit and consequently have shown lack of capacity to handle this growing burden of CVDs. Hence, the necessity to find a primary preventive measure that will reduce the impact of CVDs on the lives of people in the society.

Many studies in Nigeria have focused on assessing the burden of CVD risk factors but not the risk of future cardiovascular events [18, 19]. However, information on risk factors’ prevalence alone is inadequate to provide needed knowledge on the risk of future cardiovascular events. Furthermore, for implementation of cost-effective interventions to prevent CVDs, it is essential to be equipped with information on the proportion of people in low, intermediate or high cardiovascular risk strata. This stratification affords the clinicians and non-clinician health workers the opportunity of prioritizing interventions by allocating resources to those with high total cardiovascular risk. Presently, there is scarcity of data on this in Nigeria. Therefore, this study determined the pattern and factors associated with the risk of future cardiovascular events with a view to identifying patients who are asymptomatic but at high risk of developing CVDs.

## Methods

### Study location

The study was conducted among local government workers in selected local government areas (LGAs) in Oyo State. Oyo state is situated in the South-western part of the country. The state has a projected 2019 population of 8, 635, 793 using a growth rate of 3.4% and 2006 population figure as the baseline [20]. Oyo State is mainly inhabited by the Yoruba ethnic group who is primarily agrarian but has a predilection for living in high-density urban centres where some are traders, artisans and civil servants. The civil servants and majority of the people are involved in trading activities which are largely sedentary. In Nigeria, the ratio of a cardiologist to population is 1: 581,000 [16]. There are three tertiary health facilities in the state. There are about 60 fast food joints and restaurants (registered) while many are unregistered, four stadia and three recreational facilities.

### Study design and population

This was a descriptive cross-sectional study carried out in six selected local government areas (LGAs) of Oyo State between July and September, 2017. The study population included all the civil servants working in the LGAs’ administrative headquarters (LGA secretariats) in Oyo State at the time of study. All consenting workers who had been in the service for at least one year were recruited for the study. Pregnant women, medically unfit persons with oedema, ascites or other chronic illnesses and those who could not stand straight for weight and height measurement were excluded. Also exempted from the study were the respondents with a history of CVDs.

### Sample size and sampling technique

The minimum sample size was calculated using the sample size formula for estimating single proportion. Based on the previous study on hypertension in Delta State [21], a prevalence of 21.0% was used and the margin of error was set at 5%. A non-response rate of 10% was envisaged among our respondents and adjustment for this was made to arrive at a minimum sample size of 260. A multi-stage sampling technique was used. Stage one: Two LGAs were selected from each of the list of the LGAs in each of the three senatorial districts by ballot making 6 LGAs in all. Stage two: Each LGA headquarters has 8 departments and 4 departments were selected from each of the 6 selected LGAs using ballot technique. The number of civil servants interviewed was proportionately allocated to the LGAs. Stage three: The respondents were selected using systematic sampling and this was achieved with the aid of staff nominal list retrieved from the selected LGAs.

### Research instruments and data collection methods

A semi-structured interviewer-administered questionnaire adapted from WHO STEPS instrument [22] was used to collect information about respondents. Other questions developed from previous local studies were added to supplement WHO STEPS questionnaire so as to reflect local context [23, 24]. Questionnaire was used to collect data on socio-demographic characteristics, knowledge of cardiovascular risk factors, behavioural risk factors and medical history among the local government workers**.** Anthropometric measurements were carried out for physical assessments using stadiometer (SECA 213 Height Measure, Leicester, UK) to measure height to the nearest 0.1 m, digital bathroom weighing scale (SECA Clara 803 weight Scale, GmbH & Co, Germany) for weight measurement and non-elastic tape measure (Goldfish brand) was used to measure waist and hip circumference**.** All measurements were carried out in line with recommended standard protocols [25].

The respondents’ blood pressure was measured with OMRON 2 digital sphygmomanometer and this was done on the left arm having had at least 10 to 15 min of rest while respondents were sitting down. The cuffs were applied evenly and closely around the bare upper arm, with the lower edge 2.5 cm above the cubital fossa. The cubital fossa was approximately at heart level. The recorded blood pressure was an average of two measurements taken 10 mins apart**.**

The day preceding the study, text messages were sent to the respondents to remind them to fast overnight for the fasting lipid analysis. Ten (10) mls blood samples were collected on the spot and divided into lithium heparinized and flouride oxalate bottles from participants after an overnight fasting (8-12 h). Standard infection prevention procedures were applied in collecting blood samples from participants. The samples collected were centrifuged at 5000 g for 10 min and the plasma obtained was stored at − 20 °C in cryovials until assayed. The lipid profile was estimated by enzymatic techniques with Randox Kits using Semi-autoanalyzer (Clinical Chemistry Analyzer, HA-1900 by HAWSDEY). Fasting plasma glucose was estimated by the Glucose Oxidase method using Randox Kits. Data on 10-year risk of CVDs were captured with the WHO/ISH prediction chart [26] which makes use of age, sex, smoking status, diabetes status, systolic hypertension and total cholesterol.

Nine Community Health Extension Workers (CHEWs) and nurse/midwives who were primary healthcare staff were recruited and trained to assist in data collection. They were trained for 2 days for 3 h daily by the principal investigator on questionnaire administration and blood sample collection for lipid profile analysis and blood glucose estimation. The training involved practical demonstrations on the use of the data capturing tools. Lipid profile analysis was carried out by chemical pathologists in LAUTECH Teaching Hospital, Ogbomoso, Oyo State. The instrument was pretested among workers in local government areas different from the ones used for the main study. The pretest helped to assess the appropriateness of the questions in eliciting responses from the participants. Ambiguous questions were either removed or replaced in line with study objectives.

### Measurement of outcome variables

Hypertension was defined in a respondent as one with a history of hypertension diagnosed by a physician, was using anti-hypertensive drugs, or had a systolic blood pressure (SBP) ≥140 mmHg and/or a diastolic blood pressure ≥ 90 mmHg [27]. A respondent was considered to have diabetes mellitus if he or she had been previously diagnosed by a physician, was using blood sugar lowering medications, or had a fasting blood sugar measurement of > 126 mg/dL [28].

Obesity was defined as a BMI of ≥30 kg/m^2^. Tobacco use was categorized as ever smoked and non-smokers. The subjects who engaged in leisure-time physical activity (walking, fitness training and sports) for greater than or equal to three times per week of 30 min per occasion were classified as physically active [29]. Visceral obesity was defined as waist circumference greater than 88 cm in females and greater than 102 cm in males [30] while the Waist-hip ratio (WHR) was considered to be abnormal if it is greater than 0.85 in female and 0.90 in male [30].

The 10-year risk of fatal/nonfatal cardiovascular events was calculated using the World Health Organization/International Society of Hypertension (WHO/ISH) risk charts calibrated for use in Region of Africa, sub-region D (AFR D) [31]. Low, moderate and high CVD risks were defined as the presence of the estimated 10-year CVD risk of < 10, 10% - < 20% and ≥ 20% respectively.

### Data analysis

The data were checked daily on the field and Statistical Package for Social Sciences (SPSS) version 25 (SPSS Inc., Chicago, IL, IBM Version) was used for entry and analysis. Univariate analysis of all the variables measured was first carried out**.** Data were presented using frequency distribution tables and charts. Association between 10-year risk for CVDs and other categorical variables was assessed using chi square. For every cell with an expected value less than 5, Fisher’s Exact Test was used to determine the statistical significance. In the multivariate analysis, stepwise model of binary logistic regression analysis was done to determine the predictors of future CVD risk. Variables imputed into the logistic model were selected based on their level of significance during bi-variate analysis. Adjusted odds ratio and 95% confidence interval were obtained to identify determinants of 10-year risk for CVDs. The primary outcome measure of the study was 10-year risk for CVDs and re-categorized into high or low risk of developing cardiovascular events within 10 years using WHO/ISH prediction chart. Re-categorization was done by merging those with moderate and high risk into high risk. Respondents’ knowledge of CVD risk factors was tested with 10 questions and total obtainable score was 10, score above or equal to mean score (6) was categorized as good knowledge. Level of significance was set at *p* < 0.05 for this study.

### Ethical considerations

Ethical approval for the study was obtained from the Ethical Review Committee, LAUTECH Teaching Hospital, Ogbomoso (No: LTH/OGB/EC/2016/101). Permission was obtained from the Heads of Local Government Administration of the selected LGAs. Written informed consent was obtained from study participants before they could participate in the study. Participation was voluntary and confidentiality was ensured. Anonymity was ensured by using codes rather than participants’ names as identifiers. Data were stored in a computer that was only accessible to the principal investigator. Study participants detected to have had high risk of developing CVDs within the next 10 years were appropriately counseled and referred to specialists for expertise management.

## Results

### Socio-demographic characteristics of LGA civil servants in Oyo state

The mean age of the respondents was 46.4 ± 6.7 years. The age of the youngest and oldest respondents was 28 years and 60 years respectively. Majority 210 (80.8%) of them belonging to age 40 years and above. Nearly all 257 (98.8%) the respondents were from Yoruba ethnic group and most of them were married 228 (88.1%). Proportions of male and female were almost the same. A little less than three-quarter 186 (71.5%) of the respondents practiced Christianity. More than half 151 (58.1%) of the respondents had tertiary education while only 17 (6.5%) of them had primary education. Majority 242 (93.1%) of them had spent more than 10 years in service. Majority 242 (93.1%) of the respondents received monthly income of greater than minimum wage (N18, 000) Table 1.

**Table 1:** Socio-demographic characteristics of LGA civil servants in Oyo State (*n* = 260)

Variables	Frequency	Percentage (%)
**Age**		
< 40	44	16.9
≥ 40	216	83.1
**Sex**		
Male	128	49.2
Female	132	50.8
**Religion**		
Christianity	186	1.5
Islam	74	28.5
**Tribe**		
Yoruba	257	98.8
Others	3	1.2
**Marital Status**		
Single	11	4.2
Married	229	88.1
Widow/Divorced/Separated	20	7.7
**Educational Status**		
Primary	17	6.5
Secondary	45	17.3
Tertiary	198	76.2
**Type of Marriage**		
Monogamy	216	83.1
Polygamy	44	16.9
**Number of Years in Service**		
< 10	18	6.9
≥ 10	242	93.1
**Monthly Income (N)**		
< 18,000	18	6.9
≥ 18,000	242	93.1

### Distribution of risk factors among LGA civil servants in Oyo state

The prevalence of abnormal waist circumference, ever smoked tobacco and alcohol consumption was 93 (35.8%), 15 (5.8%) and 68 (26.2%) respectively while 220 (84.6%) and 64 (24.6%) were recorded for physical inactivity and obesity respectively. Systolic hypertension, hypercholesterolaemia, low HDLc, high LDLc, diabetes and poor knowledge of CVD risk factors were found with prevalence of 77 (29.6%), 144 (55.4%), 183 (70.4%), 59 (22.7%), 49 (18.8%) and 110 (42.3%) respectively Table 2.

**Table 2:** Distribution of risk factors among LGA civil servants in Oyo State (*n* = 260)

Variables	Frequency	Percentage (%)
**Waist Circumference**		
Normal	167	64.2
High	93	35.8
**Ever Smoked Tobacco**		
Yes	15	5.8
No	245	94.2
**Ever taken Alcohol**		
Yes	68	26.2
No	192	73.8
**Physical Activity**		
Active	40	15.4
Inactive	220	84.6
**Body Mass Index**		
Underweight	5	1.9
Normal	119	45.8
Overweight	72	27.7
Obesity	64	24.6
**Systolic Blood Pressure**		
Normal	183	70.4
Systolic Hypertension	77	29.6
**Plasma Total Cholesterol**		
Desirable	116	44.6
High	144	55.4
**Plasma HDLc**		
Low	183	70.4
Normal	64	24.6
High	13	5.0
**Plasma LDLc**		
Optimal	201	77.3
High	59	22.7
**Fasting Blood Sugar**		
Normal	126	48.5
Impaired	85	32.7
Diabetic	49	18.8
**Knowledge**		
Poor	110	42.3
Good	150	57.7

### Stratification of CVD risk among local government civil servants

**Fig. 1:**
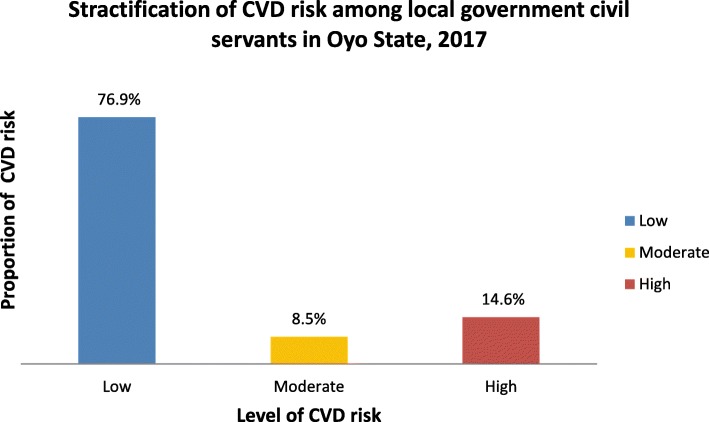
Stratification of CVD risk among local government civil servants in Oyo State

The proportion of respondents with low risk of developing cardiovascular event was 200 (76.9%) while the proportion of those with moderate and high risk of developing cardiovascular events was 22 (8.5%) and 38 (14.6%) respectively Fig. 1

### Association between socio-demographic variables and 10-year risk for CVDs among the respondents

The proportion of those with high risk of developing CVDs increased with age and was significantly higher 55 (25.5%) among respondents with age 40 years and above (*p* = 0.043). The proportion 40 (66.7%) was also significantly higher among respondents who belonged to the junior cadre (*p* = 0.003). Those respondents with higher service years (> 10 years) were found to have greater proportion 56 (93.3%) of respondents with CVD risk (*p* = 0.003). There was a significantly higher proportion 58 (23.9%) of high risk of CVDs among the respondents with secondary and tertiary education (*p* = 0.033) Table 3.

**Table 3:** Association between socio-demographic variables and 10-year risk for CVDs

Variables	10-Year Risk for CVDs (%)		***P*** value
	Low (***n*** = 200)	High (***n*** = 60)	
**Age**			
< 40	39 (88.6)	5 (11.4)	*0.043
≥ 40	161 (74.5)	55 (25.5)	
**Sex**			
Male	97 (75.8)	31 (24.2)	0.667
Female	103 (78.0)	29 (22.0)	
**Marital status**			
Single/ Widow/Divorced/Separated	26 (83.9)	5 (16.1)	0.328
Married	174 (76.0)	55 (24.0)	
**Educational Status**			
Primary	15 (88.2)	2 (11.8)	**0.033
Secondary and Tertiary	185 (76.1)	58 (23.9)	
**Staff Cadre**			
Management	110 (55.0)	20 (33.3)	*0.003
Junior	90 (45.0)	40 (66.7)	
**Number of Years in Service**			
≤ 10	17 (94.4)	1 (5.6)	**0.037
> 10	183 (75.6)	59 (24.4)	
**Monthly Income (Individual)**			
< 18,000	16 (88.9)	2 (11.1)	0.212
≥ 18,000	184 (76.0)	58 (24.0)	
**Location of LGA**			
Rural	81 (83.5)	16 (16.5)	0.052
Urban	119 (73.0)	44 (27.0)	
**Tribe**			
Yoruba	198 (77.0)	59 (23.0)	0.671
Others	2 (66.7)	1 (33.3)	
**Religion**			
Christianity	148 (79.6)	38 (20.4)	0.108
Islam	52 (70.3)	22 (29.7)	

* Significant **Fisher’s Exact Test

### Association between risk factors and 10-year risk for CVDs among the respondents

The proportion of those with high risk of developing CVDs was significantly higher 31 (31.9%) among respondents with abnormal abdominal circumference (*p* = 0.014) while it was also higher 51 (85.0%) among the respondents who added salt to the already cooked food (*p* = 0.049). Higher proportions of 53 (88.3%) and 21 (32.8%) of respondents with high risk of developing CVDs were prevalent among those who were physically inactive (*p* = 0.023) and obese (*p* = 0.033) respectively Table 4.

**Table 4:** Association between risk factors and 10-year risk for CVDs among the respondents

Variables	10-Year Risk for CVD (%)		***P*** value
	Low (***n*** = 200)	High (***n*** = 60)	
**Knowledge of Risk Factors**			
Poor	87 (79.1)	23 (20.9)	0.477
Good	113 (75.3)	37 (24.7)	
**Waist Circumference**			
Normal	138 (81.7)	29 (18.3)	*0.014
High	62 (68.1)	31 (31.9)	
**Waist-Hip-Circumference**			
Normal	62 (77.5)	18 (22.5)	0.883
Abnormal	138 (76.7)	42 (23.3)	
**Ever taken Alcohol**			
Yes	55 (27.5)	13 (21.7)	0.367
No	145 (72.5)	47 (78.3)	
**Physical Activity**			
Active	33 (16.5)	7 (11.7)	*0.023
Inactive	167 (83.5)	53 (88.3)	
**Body Mass Index**			
Non-obese	157 (80.1)	39 (19.9)	*0.033
Obese	43 (67.2)	21 (32.8)	
**Addition of salt to cooked food**			
Yes	55 (27.5)	9 (15.0)	*0.049
No	145 (72.5)	51 (85.0)	
**Eating of snacks or fast food in a week**			
≤ 3	184 (92.0)	59 (98.3)	**0.048
> 3	16 (8.0)	1 (1.7)	

* Significant **Fisher’s Exact Test

### Predictors of 10-year risk for CVD among the LGA civil servants

The binary logistic regression analysis shows that the age ≥ 40 years was associated with the higher likelihood (2.6) of developing CVD within the next 10 years compared with age < 40 years. (aOR; 2.6, CI; 1.34–8.54). Also the respondents in the management cadre were associated with the higher likelihood (3.8) of developing CVD within the next 10 years compared with those in the junior cadre categories (aOR; 3.8, CI; 1.52–9.64). The respondents with general obesity was associated with the higher likelihood (5.0) of developing CVD within the next 10 years compared with underweight respondents (aOR; 4.79, CI; 1.18–120.10) while those with central obesity was associated with the higher likelihood (2.8) of developing CVD within the next 10 years compared with those without central obesity (aOR; 2.8, CI; 1.32–5.20). Physical inactivity was associated with the higher likelihood (2.4) of developing CVD within the next 10 years compared with those who were physically active Table 5.

**Table 5:** Predictors of 10-year risk for CVD among the LGA civil servants in Oyo State

Variables	Odd Ratio	95% Confidence Interval		***p***-Value
		Lower	Upper	
**Age**				
< 40	1			
≥ 40	2.6	1.34	8.54	*0.043
**Staff Cadre**				
Junior	1			
Management	3.8	1.52	9.64	*0.005
**Educational Status**				
Primary and below	1			
Secondary and Tertiary	0.5	0.56	4.05	0.496
**Number of Years in Service**				
≤ 10	1			
> 10	0.6	0.171	1.89	0.358
**Waist Circumference**				
Normal	1			
High	2.8	1.32	5.20	*0.046
**Physical Activity**				
Active	1			
Inactive	2.4	1.21	4.70	*0.012
**Eating of snacks or fast food in a week**				
≤ 3	1			
> 3	2.2	0.78	2.05	0.141
**Body Mass Index**				
Non-obese	1			
Obese	4.8	1.18	120.10	*0.034

* Significant

## Discussion

Stratifying populations into low, moderate and high cardiovascular risk using WHO/ISH prediction charts is one of the important ways of ensuring the allocation of scarce resources to reduce the magnitude of cardiovascular outcome, especially in the groups with high cardiovascular risk. This study determined the proportion of local government workers in different categories of risk levels that would develop CVDs in the next 10 years using the WHO/ISH scoring system [31]. Our study revealed that more than two-thirds of local government workers belong to a low risk category and this was consistent with 72.6% [32] reported in United States. The high prevalence of low cardiovascular risk in this study could be due to good knowledge on risk factors. However, this was lower than 83.0% [33], 86.4% [34] and 92.6% [35] documented in India, Nepal and Saudi Arabia respectively. The low prevalence recorded in this study could probably be attributed to different study populations, especially in age distribution. In our study, the mean age was 46.4 (± 6.7) years while the mean ages in the above studies were 54.2 (±11.1) years and 53.5 (±10.1) years.

Our study also revealed 8.5% of respondents were in moderate risk of developing CVDs in the next 10 years and this was in agreement with 7.9% reported in Angola [36]. However, it was lower than 15.5 and 31.1% documented in U.S [32] and a rural community in Nigeria [37] respectively. This finding signifies the proportion of local government workers who need individualized lifestyle interventions and perhaps drug treatment considerations.

The proportion of respondents with high risk of coming down with CVDs was 14.6% and this is suggestive of those in critical need of pharmacological interventions. This proportion needs not only intensified lifestyle modification but drugs inclusion is a must in order to avert the impending CVDs. With respect to other similar studies, the prevalence of high risk of developing CVDs found in this study was in agreement with that of India (13.2%) [38] and the United States (15.5%) [39]. However, it was higher than those of rural India (10.2%) [15], Sri Lanka (2.2%) [40] and Cambodia (10.4%) [41] and lower than the Mongolian (33.3%) and Malaysian (20.8%) [41] studies. The implication of this finding is that more people will be in need of drug treatment which is probably outside their financial capacities; hence, there is an urgent need to put primary preventive measures on ground to reverse this situation.

The higher proportion recorded in our study could result from population of civil servants who are viewed as susceptible to CVD risk factors [42]. This could also result from high prevalence of systolic hypertension, total serum cholesterol, obesity and physical inactivity recorded among the study participants. However, the lower prevalence reported in our study compared with Mongolian and Malaysian studies could result from older population and a higher life expectancy at birth [43]. This is corroborated by the findings in Indonesia that increasing life expectancy and urbanization are major determinants of CVDs in developing countries [7].

Furthermore, the prevalence of high cardiovascular risk is equally lower than what was reported by Emerole in the tertiary institutions in Nigeria [44]. This lower prevalence could probably result from low socio-economic status of the respondents compared with what obtains in the university settings, since poverty has been found to be associated with a higher risk of heart-related mortalities [45].

Age was found to be one of the determinants of 10-year risk of CVDs. Respondents with age greater than 40 years were about 3 times associated with the higher likelihood of developing CVD compared with those in the age 40 years and below. This could be reasoned from the fact that those above 40 years of age belonged to the high income earners and they were mainly from the management cadre and this has been found to be associated with increased incidence of CVD risk factors [42, 46]. This is corroborated by a study in Nigeria where a higher risk of CVDs among high income earners was documented [23]. Arising from this finding, those respondents in this age category should be prioritized for screening of CVD risk factors with possible pharmacological interventions.

It was also reported from our study that respondents from the management cadre were about 4 times associated with the higher likelihood of developing CVD within the next 10 years compared with their junior cadre staff counterparts and this was consistent with a study in Sri Lanka where a respondent’s grade was found to be a stronger predictor of his subsequent cardiovascular events [47]. Evidence from our study in support of this was the higher significant difference in obesity between management (53.2%) and junior (22.4%) cadre staff.

In addition, our study also reported obesity as a determinant of CVDs as it confers about 5 times likelihood of developing CVDs compared with non-obese respondents. This is not surprising because obesity has been reported to be strongly related to major cardiovascular risk factors such as raised blood pressure, glucose intolerance, type 2 diabetes and dyslipidaemias [28]. The increased obesity noticed in this study, may generally reflect the nutritional transition being reported in developing countries, where lifestyle changes in the direction of high energy diet and sedentary habits are taking an upward turn [48]. This may be the probable reason why the prevalence rate of obesity that was reported in this study almost doubles the findings by Amole [24] in the same Oyo State 6 years ago. However, it may just be a reflection of the socio-economic status of the different study areas [23]. If higher risk of CVDs is to be reduced, therefore education of people on the risk factors for obesity and weight reduction through non-pharmacological means will be imperative.

Respondents with abnormal waist circumference were about 3 times more likely to come down with CVDs than those with normal values. Arising from this fact, it is recommended that waist circumference should be a routine measure in clinical practice to characterize health risk and of equal importance, to follow the success of strategies designed to reduce obesity and related co-morbid conditions. Those who were physically inactive were 2.4 times associated with the higher likelihood of developing CVD compared with respondents who were active. The physical inactivity amplifies the effects of other risk factors like hypertension, triglycerides, diabetes and obesity which in turns increases the risk of CVD [49]. This is in agreement with a cross-sectional study of middle-aged premenopausal women where physical activity was seen to significantly lower blood pressure, cholesterol and triglycerides [50]. As it is important for physical exercise to be encouraged by the government, so also is the need for employers to allow some time-out for physical activities among those whose work remains largely sedentary. This will help to prevent the rising prevalence of physical inactivity noticed in this study.

### Study limitation

Part of the information obtained for the study was through a questionnaire and as such the study was limited by the information supplied by the respondents as some of them found it difficult to reveal some of their attributes. However, the importance and benefits of giving correct information was explained to them in order to reduce the effects of not supplying correct information.

## Conclusion

The use of the WHO/ISH prediction charts in stratifying local government civil servants into low (< 10%), moderate (10–20%) and high (> 20%) level of cardiovascular risk is one of the crucial steps to identify and reduce the population eligible for drug intervention with a view to reducing the magnitude of cardiovascular events. Among the civil servants in Oyo State, about one-sixth (14.6%) of them will probably require more than lifestyle modification in the prevention and control of future cardiovascular events. Lifestyle modification and early detection of risk factors through awareness and regular screening programmes for those with high CVD risk is therefore recommended.

## Supplementary information

**Additional file 1.**

## Data Availability

Upon request, we can offer onsite access to external researchers to the data analyzed at the department of Community Medicine, LAUTECH Teaching Hospital, Ogbomoso, Nigeria. To do so, Dr. Olaniyan Akintunde Babatunde should be contacted.

## Abbreviations

LGAs: Local government areas
CVDs: Cardiovascular diseases
CHEWs: Community health extension workers
LAUTECH: Ladoke Akintola University of technology
LTH: LAUTECH teaching hospital
WHO: World Health Organization
ISH: International Society of Hypertension
LDLc: Low density lipoprotein cholesterol
HDLc: High density lipoprotein cholesterol
BMI: Body mass index
