# Supplementary material for: 10-year risk for cardiovascular diseases using WHO prediction chart: findings from the civil servants in South-western Nigeria
Source: BMC Cardiovasc Disord. 2020 Mar 31;20:154. doi: 10.1186/s12872-020-01438-9 (PMC7110661; doi:10.1186/s12872-020-01438-9)
Supplement: Supplementary file 1 — Additional file 1. [file 12872_2020_1438_MOESM1_ESM.docx]

**APPENDIX I**

**QUESTIONNAIRE**

**10-year risk for cardiovascular diseases using WHO prediction chart: Findings from the civil servants in Southwestern Nigeria**

**Participant Identification Number └─┴─┴─┘**

**Section A Socio-demographic data.** Tick appropriate answer or fill in the gap

1. Sex Male[ ] Female[ ]

If female, state date of your last menstruation………………….

1. Age last birthday└─┴─┘Years
2. What is your religion? Christianity [ ] Islam [ ] Others specify ____________
3. What is your tribe? Yoruba [ ] Hausa/Fulani[ ] Igbo[ ] Others specify __________
4. What is your marital status? Single [ ] Married [ ] Widow [ ] Divorced [ ] Separated [ ] Cohabitating[ ] Others [ ]
5. What is your highest educational qualification? Primary [ ] Secondary [ ]

Tertiary [ ]

1. How many years have you spent in service? ____________
2. What is your grade level? ____________
3. What is your monthly income?..........................................

**SECTION B: KNOWLEDGE OF CARDIOVASCULAR RISK FACTORS**

1. **Assessment of knowledge of cardiovascular risk Factors**

|  |  | Yes | No | I don’t know |
| --- | --- | --- | --- | --- |
| a | If you have a family history of heart disease you are at risk for developing heart disease |  |  |  |
| b | The older a person is, the greater their risk of having heart disease |  |  |  |
| c | Smoking is a risk factor for heart disease |  |  |  |
| d | High blood pressure is a risk factor for heart disease |  |  |  |
| e | High cholesterol is a risk factor for developing heart disease |  |  |  |
| f | Eating fatty foods does not affect blood cholesterol levels |  |  |  |
| g | Being overweight increases a person’s risk for heart disease |  |  |  |
| h | Regular physical activity will lower a person’s chance of getting heart disease |  |  |  |
| I | Diabetes is a risk factor for developing heart disease |  |  |  |
| J | A person who has diabetes can reduce their risk of developing heart disease if they keep their blood sugar levels under control |  |  |  |

**SECTION C: CARDIOVASCULAR RISK FACTORS (BEHAVIOURAL)**

**Smoking/Tobacco Use**

1. Have you ever smoked cigarette/use any **tobacco products**? Yes [ ] No [ ] If **NO, go to question 14**
2. When last did you smoke? [ ] days
3. How long have you been smoking?--------------/-------------months/years

**Alcohol consumption**

1. Have you ever consumed alcohol? Yes [ ] No [ ]

(I have never had a standard alcoholic drink in my life). **Skip to 18**

1. Did you take alcohol in the past 30 days? Yes [ ] No [ ]
2. How often do you drink alcohol? _____________
3. On an average, how many bottles of beer/glass of alcoholic wine/shot of gin or whisky do you have in a day?____________

**Physical activity**

1. Does your work schedule involve vigorous-intensity activity that causes large increases in breathing or heart rate like [carrying or lifting heavy loads, digging or construction work] for at least 20 minutes continuously?

Yes ( ) No ( ) **If NO go to 21**

1. In a typical week, on how many days do you do vigorous-intensity activities as part of your work? Number of days └─┘
2. How much time do you spend doing vigorous-intensity activities at work on a typical day? Hours : minutes └─┴─┘: └─┴─┘
3. Does your work involve moderate-intensity activity that causes small increases in breathing or heart rate such as brisk walking [or carrying light loads] for at least 30 minutes continuously? Yes( ) No( ) **If NO go to 24**
4. In a typical week, on how many days do you do moderate-intensity activities as part of your work? Number of days └─┘
5. How much time do you spend doing moderate-intensity activities at work on a typical day? Hours : minutes └─┴─┘: └─┴─┘

**SECTION D: CARDIOVASCULAR RISK FACTORS**

**Overweight/Obesity**

1. Height in Meters (m) └─┴─┴─┘. └─┘
2. Weight in Kilograms (kg) └─┴─┴─┘.└─┘

**Blood Pressure**

1. Systolic ( mmHg) └─┴─┴─┘ Reading-1 systolic └─┴─┴─┘ Reading 2

Diastolic (mmHg) └─┴─┴─┘ Diastolic└─┴─┴─┘

**Fasting Blood Glucose and Blood Lipids**

1. During the past 12 hours have you eaten or drunk anything? Yes[ ] No [ ]
2. Fasting blood glucose └─┴─┘. └─┴─┘ *mmol/l or mg/dl*
3. Total cholesterol└─┴─┘. └─┴─┘ *mmol/l or mg/dl*
4. LDL Cholesterol└─┴─┘. └─┴─┘ *mmol/l or mg/dl*
5. Triglycerides└─┴─┘. └─┴─┘*mmol/l or mg/dl*
6. HDL Cholesterol└─┴─┴─┘.└─┘*mmol/l or mg/dl*

**THANK YOU FOR PARTICIPATING**
